# Ambulance clinicians’ understanding of older patients’ self-determination: A vignette study

**DOI:** 10.1177/09697330231196230

**Published:** 2023-09-15

**Authors:** Anna Bennesved, Anders Bremer, Anders Svensson, Andreas Rantala, Mats Holmberg

**Affiliations:** 4180 Linnaeus University; Region Kronoberg; 4180 Linnaeus University; 4180 Linnaeus University; Region Kronoberg; 5193 Lund University; Region Skåne; 4180 Linnaeus University; Uppsala University; 6140 Region Sörmland

**Keywords:** Ambulance clinicians, autonomy, decision-making, ethics, older patient, self-determination

## Abstract

**Background:**

Older patients are often vulnerable and highly dependent on healthcare professionals’ assessment in the event of acute illness. In the context of ambulance services, this poses challenges as the assessment is normally conducted with a focus on identifying life-threatening conditions. Such assessment is not fully satisfactory in a patient relationship that also aims to promote and protect patient autonomy.

**Aim:**

To describe ambulance clinicians’ understanding of older patients’ self-determination when the patient’s decision-making ability is impaired.

**Research design:**

A qualitative design with an inductive approach, guided by descriptive phenomenology.

**Participants:**

In total, 30 ambulance clinicians, comprised of 25 prehospital emergency nurses, 1 nurse and 4 emergency medical technicians participated in 15 dyadic interviews.

**Ethical considerations:**

The research was conducted in accordance with the Declaration of Helsinki, and permission was granted by the Swedish Ethical Review Authority.

**Findings:**

The findings are presented in two themes: (1) *Movement between explicit and implicit will*; and (2) *Contradictions about the patient’s best interests.* The clinicians’ interpretations are based on an understanding of the patient’s situation using substitute decision-making in emergency situations and conversations that reveal the patient’s explicit wishes. Sometimes the clinicians collaborate to validate the patient’s implicit will, while they at other times subordinate themselves to others’ opinions. The clinicians find themselves in conflict between personal values and organisational values as they try to protect the patient’s self-determination.

**Conclusion:**

The results indicate that older patients with an impaired decision-making ability risk losing the right to self-determination in the context of ambulance services. The clinicians face challenges that significantly affect their ability to handle the older patient’s unique needs based on a holistic perspective and their ability to be autonomous.

## Highlights

• The promotion of patient autonomy is ethically challenging in ambulance service
• ACs experience conflicts between personal and organisational values
• ACs lack a mandate to bridge the gap between guidelines and the patient’s situation
• ACs dealing with paternalism are hampered by a fear of making wrong decisions
• ACs find it difficult to care for each patient based on his/her unique values

## Introduction

Older patients are often vulnerable and highly dependent on healthcare professionals’ assessment in the event of acute illness. Compared to younger patients, they are more often fragile, vulnerable and often suffer from multiple illnesses. In the context of ambulance services (AS), this poses challenges as the ambulance clinician’s (AC) assessment is normally conducted with a focus to identify life-threatening conditions and not on promoting and protecting patient autonomy. When the older patient also has an impaired decision-making ability, the risk that the patient’s mental, physical and existential needs are being overlooked increases, which means ethical values may not be respected. This study focuses on ACs’ understanding of older patients’ self-determination when the patient’s decision-making ability is impaired.

## Background

Due to an increasingly ageing population, the World Health Organisation has issued recommendations on the urgency to meet the needs of older patients and reduce their dependency.^1^ Currently, approximately two million people in Sweden are over 65 years of age (1.56 million in aged 65–70 years and 0.6 million in aged >80 years).^2^ Approximately 80,000 older Swedes live in nursing homes, which means most live at home.^3^ Older patients have an increased risk of multi morbidity, which can be assumed to increase the need for ambulance service.^4^ In this context, older patients are difficult to assess, not only medically, but also psychologically, socially and existentially – partly due to the patient’s increased risk of impaired ability for self-determination.^5^ With increasing age inevitably follows an increased population of frail older people and thus an increased risk of reduced autonomy.^6^

Sweden demonstrates a clear trend in reducing hospital beds even though the population is steadily increasing; yet, the Swedish organisation for municipal and regional healthcare estimates 3200 more hospital beds will be needed in the next 10 years, based solely on demographic changes.^7^ Consequently, this demographic change is also reflected in the ambulance service, showing that 59.9% of patients were aged 70 years or older during 2017 and 2018.^8^ There are statistics demonstrating that the number of people seeking emergency care has been steadily increasing since 2015.^9^

Within the ambulance service, the ACs are exposed to the unexpected and unknown, where the initial assessment may rapidly change at the scene depending on the patient’s condition.^10^ Their assessments, largely governed by guidelines and protocols,^11^ is normally conducted using structured assessment procedures with the primary focus to identify life-threatening conditions and provide life-saving measures.^12^ However, attention should also be paid towards the relationship with the patient,^13^ not least to protect important ethical values.^14^ Autonomy is such an important ethical value, within which the patient’s self-determination is central.^15^ Autonomy – as an ethical principle – means that all persons should have the power to make rational decisions and moral choices because all persons have inherent and unconditional values. This means that all persons should be allowed to exercise their capacity for self-determination.^16^ It has been shown that ethical conflicts arise as a result of the ACs being unable to communicate with the patient and uncertainty surrounding the patient’s decision-making ability. Older patients may have an impaired decision-making capacity or a reduced ability to participate in decisions concerning their own care, making it more difficult for the ACs to respect the patient’s autonomy due to uncertainty about the patient’s wishes and value preferences. This results in an increased risk of value conflicts.^14^

Self-determination is also described as ‘the act or power of making one’s own choices or decisions’.^17^ From that perspective, self-determination can be described as the ability to make decisions based on given alternatives.^18^ Exercising self-determination means taking a stand on those small and large decisions in everyday life that affect the individual. However, exercising one’s right to self-determination does not exclude the possibility of dependency on others for day-to-day living.^19^ Hence, self-determination can result in ethical challenges when people with limited ability must choose and act based on their own choices.^20,21^

In the context of ambulance service, older and frail patients have complex care needs, which places great demands on the ACs’ ability to identify and manage these needs while respecting the patient’s self-determination and considering their decision-making ability. Hence, the ACs’ attention to the patient relationship, with the aim of promoting and protecting patient autonomy, is central. Therefore, the aim of this study is to describe ACs’ understanding of older patient’s self-determination when the patient’s decision-making ability is impaired.

## Methods

The study was guided by the principles of descriptive phenomenology.^22^ An inductive descriptive qualitative design was used with dyadic interviews^23^ employing a case vignette technique.^24^ The study was performed in line with the Consolidated criteria for reporting qualitative research (COREQ) checklist (Appendix 1).^25^

### Participants

Purposeful sampling was applied,^26^ and the inclusion criterion was a clinically active AC. All employed ACs (*n* = 63) in the regional ambulance districts were informed of the study both verbally and in writing by the first and third author at their workplace. In addition, the unit manager informed the ACs verbally and in writing at daily staff meetings. The participants contacted the researchers to express interest in participating. In total, 30 ACs agreed to participate (Table 1).

**Table 1.** Demographics of the participants (*N* = 30).

	RN (*n* = 26)	EMT (*n* = 4)
Women, *n*	11	0
Men, *n*	15	4
Age, *median years (range)*	39 *(30–56)*	57 *(47–65)*
Work experience^a^, *years (range)*	12 *(1–28)*	24 *(14–40)*

^a^Work experience within the ambulance service.

The participants were assigned to constellations of dyadic interviews (*n* = 15) based on a convenience principle. The pairs consisted of work partners and colleagues. To mimic a common situation where the ACs work with different partners, they were divided according to how they were placed on the same shift by the manager. In some interviews, one person was present during a work shift and the other one appeared from outside the working shift. All ACs who agreed to participate followed through. The interviews were performed by the first author during work-shifts at the workplace, but not while the participants were operationally on call for ambulance assignments.

### Data collection

Data were collected between December 2019 and February 2020 through dyadic interviews^23^ employing a case vignette technique. Dyadic interviews were chosen because of the interaction that occurs between the participants in order to stimulate reasoning that would otherwise not have been recognised or remembered.^27^ Dyadic interviews are more in line with how the study participants usually work, as the case vignette should illustrate a commonly occurring scenario in the ambulance service. Case vignettes are short descriptions of a situation with specific circumstances, on which the interviewees are invited to reflect.^24^ The vignette method is, for example, relevant in studies of professionals’ actions as it enables one to gain knowledge about their ideas, explanations, norms, values and ethical sensitivity.^28^ All interviews were based on an identical vignette, enabling the participants to have their options and assessments defused on a topic that is considered sensitive.^29^ The case vignette and interview guide were pilot tested using students in the specialist nursing programme in prehospital emergency care (Appendix 2).

The construction of the vignette was based on literature reviews, literature on methods and clinical experiences in an ongoing critical review including all authors. The dyadic interviews were conducted based on a three-step case vignette, containing an ethical dilemma that the participants were encouraged to assess.^30^ The vignette was based on a care situation that explicitly or implicitly actualises the ethical issues and values it intends to illustrate.

The interviews were performed in relation to a joint structure and started with one initial open-ended question after the participants read the first part of the vignette: ‘How do you understand the patient’s decision-making ability?’ Follow up questions were then collected as field notes in order to elaborate further, for example, ‘Can you please describe more?’ Part two and three of the vignette were given to the informants when the interviewer recognised that the discussion did not provide additional information.

The audio recorded interviews lasted between 35 and 72 min (average 61 min) and were transcribed verbatim using a professional transcriber.

### Data analysis

The data was analysed using reflexive thematic analysis^11^to identify patterns of meaning.^22^ The first author began reading the interviews several times in their entirety to obtain an overall impression of their content and become familiarised with the data. In accordance with reflexive thematic analysis data was extracted, marked and labelled with temporary and tentative codes corresponding to the study aim.^31,32^ The labelling and meaning of all initial codes were repeatedly compared, collated and validated against the transcripts. Through the 353 codes that were constructed, the process of identifying broader patterns of latent meanings was continued. Initially, the codes were collated into three potential themes with six sub-themes. The themes were critically reflected upon and amended several times. This process of identifying and clustering themes was repeated until a sense of significance – within and between – the themes were reached. This process resulted in two themes with five sub-themes that was labelled. Interpretations were validated by returning to the original transcribed data, where the first and last author critically discussed their meaning. The sub-themes and themes were formulated into text to determine a logical structure across the themes. In line with descriptive phenomenology, the analysis was guided by the methodological principles of openness, questioning pre-understanding and a reflective attitude. The phenomenon was in focus and an approach for openness to the lifeworld was adopted. The authors also had an open stance sensitive to the expression of experiences. Throughout the research process and the analysis the researchers handled previous assumptions by questioning their pre-understanding. The researchers aspired to a reflective approach throughout the process to become more aware of their assumptions and reflect on the context.^22^

### Ethical considerations

The study was conducted according to the principles of the Declaration of Helsinki.^33^ Permission from the Swedish Ethical Review Authority was granted prior to the study (No. 2019-02127). All participants signed a written consent form prior to the data collection and were informed that their participation was voluntary and that they could withdraw at any time without providing a reason.

## Findings

**Figure 1.**
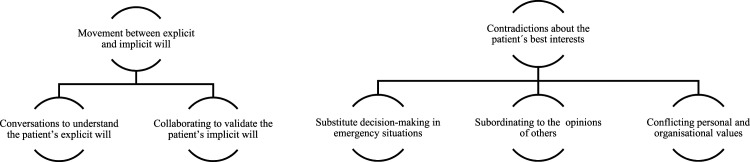
Themes and sub-themes.

The findings consists of two main themes comprising five sub-themes (Figure 1).The findings indicate challenges substantially affecting the ACs’ possibility to handle the older patient’s unique emergency needs and self-determination as a goal of satisfying the patient’s will. In the meeting with the patient and the cooperation with family members, assistant nurses and other healthcare professionals must understand the patient’s values and preferences. Additionally, the ACs make judgements based on experience as well as clinical, medical and caring knowledge, which may both maintain or undermine the ability to support the patient’s self-determination.

### Movement between explicit and implicit will

A movement between explicit and implicit will means to use the conversation to understand the patient’s explicit wishes and collaborate to validate their implicit ones. However, the assessment of the patient’s current condition in relation to their baseline condition must be performed without background information. Collaboration with assistant nurses and healthcare professionals that have knowledge of the patient thus help the ACs to handle the patient’s self-determination.

### Conversations to understand the patient’s explicit will

Based on the vignette case, the ACs perceived the conversation with the patient as part of understanding the whole by assessing the patient’s cognitive status. This is part of the understanding of his/her ability to self-determine. When the patient participates actively and with credibility in the conversation, the AC can ensure that information is understood correctly. If the patient presents his/her will in an adequate way, this limits the doubts compared to vague and diffuse expressions. However, the conversation is difficult if the patient lacks communicative abilities, which challenge the understanding of their self-determination.

‘No, no, no, I don’t want to!’/…/Of course, for me it says that she wants to be [left alone]. She doesn’t want to... ‘No, I don’t want to!’ But of course, it can be interpreted as, what is it that she doesn’t want? (Interview 2)

The ACs adapted to the patient’s ability to converse by asking simple questions. Hence, a need to understand the patient’s normal conversation abilities is highlighted. Without proper access to background information, the current situation is the base for the assessment. The conversation is used to judge the patient’s ability to express his/her explicit will. When the AC perceives that the patient’s ability to communicate is impaired, they suspect an acute deterioration. In such situations, self-determination is not considered while the ACs act according to what they consider is the patient’s best interests. However, the conversation has a secondary meaning when obvious cognitive impairment such as dementia and psychotic conditions are present. In such situations, the patient’s ability to self-determine cannot be interpreted, except when clearly documented beforehand.

### Collaborating to validate the patient’s implicit will

Based on the case and personal experiences, the ACs perceived that the understanding of patients’ self-determination is promoted through collaboration with family members, assistant nurses and healthcare professionals that have previous knowledge of the patient. Through information from others, ACs try to compare their statements with statements from the patient to find out if he/she has an implicit will that remain hidden. Such knowledge is considered necessary to be able to determine the patient’s normal condition.

On the one hand, we asked the family members, a bit on the side: ‘How much can you trust what she says, and how perceptive is she?’ And we asked the home service the same, and ‘No, but she’s probably a bit forgetful, but still... she says she doesn’t want that anyway, well, that’s the way it is... then she doesn’t want that’. (Interview 2)

Assistant nurses and healthcare professionals with insight into the patient’s situation are perceived as able to interpret the implicit communication such as facial expressions, glances, or unspoken words. To that extent, collaboration promotes the ACs’ understanding of the patient’s self-determination when decision-making is judged as insufficient. This adds further to the general picture of the patient’s situation. Other healthcare professionals may also contribute with additional medical expertise, access to medical records and support in continued treatment. Through collaboration, the ambition is to clarify the patient’s own will to support the continued care, even though their decision-making ability is judged to be temporarily impaired.

### Contradictions about the patient’s best interests

When ACs’ point of view about the patient’s best interests are overruled, it makes them subordinate themselves to the decision-making of others. Conflicts between personal and organisational values further place ACs in moral distress. Whose will is given priority in different situations varies, that is, the power to settle the treatment plan, consideration of the patient’s self-determination and the ability to judge the patient’s capacity for decision-making are sometimes regarded as outside the ACs’ responsibility.

### Substitute decision-making in emergency situations

The vignette case gave the ACs space to reflect based on earlier experiences, indicating that they act as substitute decision-makers in situations when the patient’s decision-making ability is judged to be impaired. The patient’s physical and mental health condition is described as important for the patients’ ability to be autonomous. The patient may express a desire not to be conveyed to hospital or wait until the next day, despite failing vital parameters and having been assessed as needing emergency care. This challenges the ACs’ respect for, and understanding of, the patient’s self-determination, but it is not de-prioritised out of reluctance. Instead, the decision is based on a medical responsibility that prioritises the patient’s need of care, which at the same time raises the question of whether the decision was ethically justifiable.

But also... what gave me the right there, when she herself said she didn’t want to [come along to the hospital]? Then you can of course explain. And I told her that there is a risk that you will die at home if you don’t come with us to the hospital. And it’s a good thing to say. But in the end, then the patient really must choose for himself. (Interview 15)

In emergency situations, there is limited time which make it difficult to properly understand, handle and respect the patient’s self-determination. Vital signs are assessed based on fixed references and not in light of the unique patient in a unique situation. Considering the importance of abnormal vital signs in relation to what is normal for the unique patient. This affects the ability to respect the patient’s self-determination since abnormal vital signs according to protocols may be normal for the patient and not prompt the need for emergency care. Hence, autonomous wishes are not requested in cases with deviant vital signs if the patient does not express explicit resistance.

### Subordinating to the opinions of others

The ACs reflections, based on the vignette case, was on one hand to subordinate themselves (e.g. to physicians and family members), but sometimes they simply let others decide. They described that the will of the patients is not given priority when the patients are unable to express themselves. In such situations, often an assertive family is given priority and power to make plans for action. However, this does not always mean unreflectively agreeing with those opinions. Sometimes the ACs do not act in agreement with the family members suggestions, in situations when the ACs can be supported by written documents (e.g. end-of-life-care plan and ambulance service guidelines) or if the patient explicitly disagrees. ACs also describe how they select specific information or try to persuade the patient to find a solution that is consistent with as many of the parties’ opinions as possible.

ACs experience difficulties in contradicting a decision made by a physician. This often concerns a differing opinion regarding the patient’s best interests with respect to whether the patient should be transported to the hospital. In these cases, the physician is usually given a superior role, and the AC handles the situation according to the physician’s judgement, even when they do not agree. This means that the ACs take actions that may entail forcing the patient, which is not in line with their view of good care. The care provided is then described as abusive and lacking in respect for the patient’s self-determination as the patient’s best interest is not in focus.

No, I had two physicians that had said she had to go into the hospital. And then I followed that decision, although my whole inside was screaming that this lady should be allowed to stay in her home and she should be fine. I think it’s terrible. I think it’s like abuse!/…/This is so wrong. I don’t stand by this. It goes against every cell in my body to go through this. (Interview 7)

A hierarchical implicit order is also described in which the patient’s self-determination is subordinated to the will of others. ACs can be called by other people, which does not necessarily follow a voluntarily decision by the patient. Such pressure can cause the patient to change their perception of the need for care in order to please others, even when it is not in agreement with their own will. Thus, the patient’s self-determination is not properly valued since they do not express their will.

### Conflicting personal and organisational values

The ACs reflected about prehospital emergency care in general, based on the vignette case, and considered themselves as possessing a legal and ethical interpretive precedence when the patient lacked the ability to express their will. With an expected acute deterioration, ACs can be called even though this is a natural part of the patient’s disease. If there is lack of previous documentation, there is a risk that the ACs’ care does not apply to the patient’s will. The ACs can choose to act in agreement with the patient’s current expressions or act according to organisational values and predetermined guidelines. Personal values – one’s own moral compass – evokes a feeling of acting unworthily if interventions that solely focus on prolonging the patient’s life are prioritised – which equates to acting according to organisational values and predetermined guidelines. The ACs emphasise the importance of being able to help the patient reduce suffering and establish security for the patient and family members when life-saving measures are de-prioritised. End-of-life care is an example of when personal and organisational values can clash. Such situations are characterised by an urgent medical need for care with, for example, impaired consciousness, where the overall picture speaks more to helping the patient and family members experience a dignified death. If an assessment is not made that takes into account the bigger picture, it can mean that the suffering patient is not given the opportunity for a calm and secure end-of-life care, which creates a conflict between the ACs’ personal values and organisational values.

It can be a very vulnerable and weak person at the end of life who has been deprived of the right to decide over his own life. And so, then I am a part of reinforcing that, by ruling over that person. If you relate to yourself, how important self-determination is... Just because there’s a patient lying there and can’t fight anymore, what gives me the right to take the last thing from that patient? It’s not a feeling that makes you proud, of course. (Interview 5)

The ACs have legal obligations, and they experience a discrepancy between following these obligations while simultaneously respecting patients’ self-determination. When personal and organisational values are in conflict, the ACs’ have experience in acting more often according to what is considered correct from an organisational perspective. This is based on a fear of being reported for not having acted correctly according to ambulance service guidelines and legitimate obligations. At the same time, this generates a resignation over feeling unable to provide care with the patient’s will in focus. However, ACs with long experience will to a greater extent dare to act independently in relation to the patient’s self-determination.

## Discussion

The results indicate challenges substantially affecting the participants ability to handle the older patient’s unique needs and ability to be autonomous. Overall, the results show that impaired decision-making ability means reduced self-determination. The participants made judgements based on experience as well as clinical, medical and caring knowledge. They weigh these parts together when dealing with the older patients’ self-determination to assess their decision-making capacity. When the outcome of the assessment does not correlate with ambulance service guidelines or differs from assessments made by others, ethical conflicts may arise, and the patient’s self-determination risks being neglected. These conflicts risk being aggravated by the feeling of not having a mandate to bridge the gap between ambulance service guidelines and the patient’s situation.

The participants had faith in their ability to move between the patients explicit and implicit will in order to make a holistic assessment. This overall assessment can be explained by the results of previous studies on clinical reasoning, described as a process of gathering, evaluating and using available information in order to make decisions.^10,34,35^ Furthermore, previous research describe how ACs must adopt a holistic perspective of the patient’s situation to make a complete assessment.^36^ They also describe, in agreement with our results, that several components are required to be able to make a complete assessment. It requires a balance of the patients’, family members’ and other healthcare professionals’ experiences of care needs and the situation. To reach this, the AC needs to reflect on the environment surrounding the patient in order to achieve informed decisions regarding patient care and a course of action.^37^

Clinical reasoning can be assumed to be even more important in the care of older patients as it is complex and challenges assessments. The clinical picture is often more difficult to comprehend in older patients due to polypharmacy, cognitive decline or even natural bodily changes that make critical conditions hard to detect,^38^ a medical assessment is not sufficient to make an informed decision. Overall, the participants lacked a clear picture that includes physiological, psychological and existential needs to make a well-founded decision. When there are time-critical conditions, participants adopted, to a greater extent, substitute decision-making. In cases of time-critical injuries and conditions, the participants experienced ethical conflicts to a greater extent, demanding a balance between the explicit and implicit will of the patient, weighted against guidelines and medical parameters to determine whether the patient has decision-making capacity. Previous research confirms that older patients are often vulnerable and dependent, especially in the case of impaired decision-making ability, which increases the risk of important ethical values being violated.^6^ If time-critical conditions are also added to a impaired decision-making capacity or ability to participate in the decisions, the ACs ability to understand and respect autonomy is further hindered because of uncertainty about the patient’s authentic wishes and values.^14^ This can be explained with the help of clinical reasoning that describes that assessment and decision-making are continuous, intertwined, inseparable and mutually-dependent sub-processes.^37,39,40^

The results indicate that participants felt that the patient is sometimes treated according to care that is not in line with what the patient considers right. Instead, care is carried out according to the will of others and their opinion of what is right. It often involves divided opinions about whether the patient should be conveyed to hospital. Previous studies describe this as paternalism from an ethical perspective; ethical paternalism overrides the patient’s values in favour of someone else’s.^41–43^ Other studies show that ACs, to a greater extent, find it more difficult to leave the patient at home even if it is in line with the patient’s, and others’, wishes. This is because a limited amount of non-conveyance guidelines or protocols is available for specific patient populations. The non-conveyance decisions are perceived to entail a great responsibility for the AC, and there is a fear of being reprimanded if the wrong decision is made.^44,45^ It is worth taking into account that ACs often only have two different levels of care – non-conveyance and conveyance to hospital.^46^

In connection with acute illness and/or injury, the older patient’s self-determination may be temporarily impaired. On one hand, previous studies describe how healthcare professionals handle this by providing vague information to maintain the patient’s spirit. The reason was that the professionals believed that full information would do the patient more harm than good in the specific situation.^47^ On the other hand, previous research shows that paternalism can result in patients receiving too much care or care at a different level than the patient wishes. This can be defended if ACs make the assessment that the patient later, in a more stable situation, would accept the paternalistic actions. This means that the absence of paternalistic actions can have serious negative health consequences for the already vulnerable patient. Paternalism may be justified when there are good reasons to believe that the patient in the current situation is not capable of making informed decisions.^48^

Overall, this study shows that patients are at risk, or given the opportunity for paternalistic care, not because of bad intentions on the part of the participants, but due to a fear of what a wrong decision could mean for the patient and the participant as a professional. Such a fear of harming the patient has previously been described by Swedish ACs in the patient assessment process.^49^

### Methodological considerations

This study has some limitations. First, the first author, who had limited experience of interviewing, conducted the interviews. However, a pilot interview was completed for training purposes before data collection, where feedback was given afterwards by the more experienced researchers/authors. Second, all authors have prehospital experience from ambulance service and hence a pre-understanding of the studied phenomenon. On one hand, this may constitute a threat to objectivity; on the other hand, the pre-understanding can be an asset that helps researchers understand the data better.^50^ However, there were discussions between all researchers throughout the process to prevent bias resulting from unbridled pre-understanding. The findings were constantly and critically discussed between all researchers during the analysis process until consensus was reached. The analysis was continuously challenged by the authors, returning to the raw data before the final interpretation was reached. Trustworthiness was promoted by describing the research process carefully to make it possible for others to follow the process and transfer the results to other settings. The authors had a long engagement with the data analysis and applied researcher triangulation which are believed to increase credibility. The result is judged to be transferable – through thick descriptions of the participants’ experiences and the meaning of the phenomenon – to similar contexts where autonomy and self-determination are viewed as important values and where ACs have a competence and mandate similar to those in Sweden.^51^

## Conclusion

The results indicate that older patients with an impaired decision-making ability risk the right to self-determination in the context of ambulance service. The results also underline the complexity of ACs’ understanding of older patients’ self-determination, primarily based on experience and an ambition to take a holistic perspective and respect the patient’s genuine will. When older patients have an impaired decision-making capacity, their self-determination is challenged by the actions of family members and healthcare personnel; a lack of patient information; rigid guidelines; and complex care situations. This forms a breeding ground for ethical conflicts that also risk being aggravated by a lack of moral reasoning regarding how ACs can promote and protect patient autonomy by responsibly balancing a paternalistic approach and the patient’s capacity for participation. Moral courage can be a strength in the conflict between the ACs’ personal and professional values, but also a weakness if the courage lacks a clear anchoring in humans’ equal value and the right to equal care.

## Supplemental Material

Supplemental Material - Ambulance clinicians’ understanding of older patients’ self-determination: A vignette study Supplemental Material for Ambulance clinicians’ understanding of older patients’ self-determination: A case vignette study by Anna Bennesved, Anders Bremer, Anders Svensson, Andreas Rantala and Mats Holmberg in Journal of Nursing Ethics

Supplemental Material - Ambulance clinicians’ understanding of older patients’ self-determination: A vignette study Supplemental Material for Ambulance clinicians’ understanding of older patients’ self-determination: A case vignette study by Anna Bennesved, Anders Bremer, Anders Svensson, Andreas Rantala and Mats Holmberg in Journal of Nursing Ethics
