# Supplementary material for: Ambulance clinicians’ understanding of older patients’ self-determination: A vignette study
Source: Nurs Ethics. 2023 Sep 15;31(2-3):342–54. doi: 10.1177/09697330231196230 (PMC11181732; doi:10.1177/09697330231196230)
Supplement: Supplemental Material - Ambulance clinicians’ understanding of older patients’ self-determination: A vignette study [file sj-pdf-2-nej-10.1177_09697330231196230.pdf]

Appendix 2: Vignette

Alarm text:
Category 1 assignment.
Case index 1: Uncertain data.
Case index 2: Suspicion of influence on vital parameters.
Female 76 years old. 12, South High Street. Declining consciousness, according to the son on site.

Stage 1

You receive scant information on the alarm. It concerns a woman, Edith, who is 76 years old. Jack (the son) calls an ambulance because his mother with cancer is unwell.

You arrive at the address which is twelve miles from the ambulance station. Jack meets you at the door and tells you that it is urgent. His mother is not doing well at all. Inside the apartment, Avery from the home care service sits on a chair next to Edith, who is in bed. Avery moistens Edith's oral cavity with a stick.

Edith does not answer when you introduce yourself to her. From a distance you can see that she is breathing. Edith seems bothered when you put your hand on her leg and she says weakly:

- "No, no, no - I don't want to!"

You ask Jack about Ediths medical history. Jack rushes around the room and looking for Edith's things to bring to the hospital.

Stage 2

While you are examining the patient, Pamela appears. Pamela is a nurse on the palliative team. It turns out that Jack also called her when he had alerted the ambulance. Pamela looks bothered when she enters the room and asks to talk to Jack in private. Jack quickly replies:

- "I'll just pack up mom's things so that the ambulance can leave before my sister comes back. You know what a turmoil she can create. She means well, but has a hard time standing up for our mother's rights, so I felt it was time for me to take an airplane to mom and take control of the situation."

Pamela says:

- "Can you wait a moment because I need to talk to Jack?

Pamela and Jack stand in a corner and you can hear the conversation. Pamela says:

- "The goal of end-of-life care is to create the best conditions for quality of life and to relieve Edith's symptoms. A few weeks ago, she was given i.v. fluids and she could not cope with it and swelled up in her body and got shortness of breath. Edith's physician talked to her after that and they jointly decided to end her treatment. Your mother wishes to stay here and avoid any more hospital visits."

Jack replies brusquely:

- "I need to talk to the physician. Give me his number.

Stage 3

Avery, who is sitting next to Edith, gets up and tries to convince Jack that she too has heard Edith express several times that she does not want to go in an ambulance. Jack loses his patience and says to you:

- "Okay, now let’s forget what they say and do as we decided from the beginning. I haven't seen my mother for a long time, but I've known her all my life and she's always wanted to have as good quality of life as possible."

Meanwhile, Pamela has tried to call the physician in charge of the patient but it turns out that she is off duty. Instead, she speaks to a substitute physician who is available. The physician repeats what he had already said to Jack:

- "Well, like I just told to Jack, I think the patient should come to hospital. After all, she hasn't eaten or drunk for days. There is a possibility that she is dehydrated, which could explain her behavior. There's nothing to lose by coming into hospital for a checkup and giving her some fluids."

Pamela takes over the conversation and becomes increasingly clear in her tone. She says:

- "Haven't you read the note from the GP, saying that Edith wants to stay at home and not go to hospital?"

The physician answers:

- "Yes, I have seen that, but Jack is very eager to have Edith examined, and she is welcome to this department where she is well known. Unfortunately, I do not have time to make a home visit today. I'm also just a substitute physician and I still have a lot to do before I can go home. Further, I have very little background information about Edith and therefore I can't make another decision. I think it will be best for her to be taken to hospital. She'll probably be back home soon."

In all the chaos, Rosie (Edith’s daughter) enters. She is furious when she sees what has happened while she has been away. She tightens her eyes in on you and your colleague, and says:

- "This is not okay! This is not what my mother wants."
